# Behavioral and facial thermal variations in 3-to 4-month-old infants during the Still-Face Paradigm

**DOI:** 10.3389/fpsyg.2015.01586

**Published:** 2015-10-14

**Authors:** Tiziana Aureli, Annalisa Grazia, Daniela Cardone, Arcangelo Merla

**Affiliations:** ^1^Department of Neuroscience, Imaging and Clinical Science, University “G.d’Annunzio”, Chieti, Italy; ^2^Infrared Imaging Lab, Institute of Advanced Biomedical Technologies, University “G.d’Annunzio”, Chieti, Italy

**Keywords:** still-face paradigm, infant bio-behavioral responses, thermal infrared imaging, autonomic nervous system, infants’ sensitivity to interactional reciprocity

## Abstract

Behavioral and facial thermal responses were recorded in twelve 3- to 4-month-old infants during the Still-Face Paradigm (SFP). As in the usual procedure, infants were observed in a three-step, face-to-face interaction: a normal interaction episode (3 min); the “still-face” episode in which the mother became unresponsive and assumed a neutral expression (1 min); a reunion episode in which the mother resumed the interaction (3 min). A fourth step that consisted of a toy play episode (5 min) was added for our own research interest. We coded the behavioral responses through the Infant and Caregiver Engagement Phases system, and recorded facial skin temperature via thermal infrared (IR) imaging. Comparing still-face episode to play episode, the infants’ communicative engagement decreased, their engagement with the environment increased, and no differences emerged in self-regulatory and protest behaviors. We also found that facial skin temperature increased. For the behavioral results, infants recognized the interruption of the interactional reciprocity caused by the still-face presentation, without showing upset behaviors. According to autonomic results, the parasympathetic system was more active than the sympathetic, as usually happens in aroused but not distressed situations. With respect to the debate about the causal factor of the still-face effect, thermal data were consistent with behavioral data in showing this effect as related to the infants’ expectations of the nature of the social interactions being violated. Moreover, as these are associated to the infants’ subsequent interest in the environment, they indicate the thermal IR imaging as a reliable technique for the detection of physiological variations not only in the emotional system, as indicated by research to date, but also in the attention system. Using this technique for the first time during the SFP allowed us to record autonomic data in a more ecological manner than in previous studies.

## Introduction

The still-face effect is a very well known phenomenon in developmental research. It occurs when an infant is involved in the Still-Face Paradigm (SFP), an experimental procedure that was designed by Tronick et al. (1978) to determine the infant responses to the mother’s alterations in communicative signals during face-to-face interactions. According to the literature, when the mother puts on a neutral and unresponsive face following a period of spontaneous play, infant gazing at the parent, smiling and vocalizing decrease, while negative affect, as well as self-regulatory behaviors, increase. This pattern changes when the mother goes back to the usual interactive style, as the infant too resumes the communicative engagement shown before the still-face episode, although this can be interspersed with fussing and crying behavior due to the carry-over of the negative affect from the previous aversive experience (Tronick et al., 1978; Weinberg and Tronick, 1996; Adamson and Frick, 2003; Mesman et al., 2009). As a result of the wide variety of studies first reviewed by Adamson and Frick (2003), and more recently by Mesman et al. (2009), the still-face effect has proven to be very pervasive. It is found at different ages, from 1 month of life onward, for both genders, and for normative as well as at-risk subjects.

Several pivotal explanations for the still-face effect have been proposed. They refer to the violation of the infant’s expectation of the right way of interacting (Tronick et al., 1978), to the adult withdrawal of regulatory inputs (Field, 1994), or to the dyad’s failure in shared meanings (Tronick et al., 1980) as the main causal factors, thus calling into question the cognitive, affective, or intersubjective domains, respectively. Indeed, the temporary maternal unresponsiveness brought about by the experimental paradigm impacts upon different aspects of infant functioning, and therefore, the still-face effect continues to elude a complete explanation (Adamson and Frick, 2003; Frick and Adamson, 2003; Tronick, 2003), leaving any interpretation as relatively tentative (Mesman et al., 2009). However, one aspect has obtained consensus. This effect provides the best evidence of the infant’s sensitivity to interactional reciprocity. It shows that, when faced with a sudden, unexpected change in the usual way of interacting, infants react at a communicative, attentional, and emotional level, by retreating from social engagement, orienting to the environment, and becoming disturbed.

A number of studies have investigated infants’ reactions to their mother’s still-face at the physiological level, focusing primarily on the influence of the autonomic nervous system on heart functioning. According to the Polyvagal Theory (Porges, 2007), the autonomic system affects cardiac activity in two opposite directions, from slowing down the heart rate to arresting, or speeding it up for movement. In the former, the parasympathetic component of the system is active, by enhancing the vagal break from the beating heart to promote calm states. In the latter case, this component withdraws to potentiate the sympathetic component, which increases the heart rate to mobilize resources to face environmental demands. While vagal tone supports social engagement in positive situations, the withdrawal of vagal influences facilitates the disruption of that engagement and the coping with negative cues. This functioning is thought to be best defined by the amplitude of the respiratory sinus arrhythmia (RSA), a naturally occurring variation in the heart rate during a breathing cycle. This is derived from the beat-to-beat heart rate pattern (Porges et al., 1994; Moore et al., 2009) and changes in the opposite manner with respect to heart rate, i.e., it increases in positive situations and decreases in negative situations. Studies on the infant’s autonomic reactions during the SFP have been quite consistent. The heart rate increases from play to still-face episode, and decreases during the reunion episode (Weinberg and Tronick, 1996; Bazhenova et al., 2001; Haley and Stansbury, 2003; Moore and Calkins, 2004; Ham and Tronick, 2006; for a partial exception, see Conradt and Ablow, 2010). Accordingly, the RSA amplitude shows the opposite pattern (Weinberg and Tronick, 1996; Bazhenova et al., 2001; Moore and Calkins, 2004; Ham and Tronick, 2006; Moore et al., 2009). As the above findings signal the prevailing of the sympathetic component over the parasympathetic, the cardiac measures are thought to confirm the distressing nature of the paradigm. Other studies have provided consistent results, showing that cortisol, stress hormone (Haley and Stansbury, 2003), and the skin conductance response, which is a direct measure of sympathetic arousal (Ham and Tronick, 2006), increase during the paradigm shift.

However important the above studies are for an integrated bio-behavioral picture of the still-face effect, the measurement methods used to investigate autonomic reactions are disputable. They require manipulation of the infant’s body in a more or less intrusive manner, thus introducing an extraneous source of stress to an already stressful situation. Recently, thermal infrared (IR) imaging was used to collect autonomic data under more ecological conditions. Using a thermal camera located at a convenient distance from the subject, cutaneous temperature signals released by the human body can be recorded (for a recent review, see Ioannou et al., 2014) in a contact-free manner. Significant thermal variations have been revealed in humans and in primates in response to emotional or distressing conditions. Specifically, when faced with threatening, painful, frightening or frustrating stimuli, the skin temperature decreases in some facial regions, such as maxillary, nasal tip, and cheeks areas, whereas it increased in periorbital and supraorbital areas (Levine et al., 2001; Pavlidis et al., 2002; Nakayama et al., 2005; Puri et al., 2005; Merla and Romani, 2007; Zhu et al., 2007; Shastri et al., 2009; Kuraoka and Nakamura, 2011; Ebisch et al., 2012; Manini et al., 2013; Di Giacinto et al., 2014; Engert et al., 2014; Merla, 2014). The complex interactions of cutaneous heat variations that involve skin tissue, inner tissue, local vasculature, and metabolic activity, were proposed as the mechanisms that underlie the observed autonomic variations. Innervation from both the sympathetic and parasympathetic nervous systems is commonly received by all body tissues. During challenging situations, the sympathetic component causes the skin temperature to decrease, and the skin blood vessels to constrict (although smaller vasodilatory effects can also be observed; Smith and Kampine, 1990). Due to vasodilatation and the return to an internal balanced state, the opposite occurs when the environmental challenges have passed, and a gradual temperature increase results from parasympathetic restoration. As the above physiological events cause the skin temperature to fluctuate, the opportunity to observe thermal signals allows autonomic system activity to be inferred as well as the balance between the sympathetic and parasympathetic components. According to the Porge’s Polyvagal Theory, this balance is an integral component of social engagement and mediates an individual’s affective adjustment.

Due to its potential to non-invasively record physiological reactions that reflect emotional states (Engert et al., 2014), thermal IR imaging has also been used in developmental research, although rarely. Mizukami et al. (1987, 1990) observed infants aged 6 to 29 weeks when separated from their mothers. The mean temperature of the forehead dropped significantly when the infants were left alone, or when a stranger replaced the mother in the room, which suggested that the infants experienced anxiety or stress. A group of studies (Ebisch et al., 2012; Ioannou et al., 2013; Manini et al., 2013) presented 3- to 4-year-old children with the “mishap paradigm,” a procedure devised to elicit a sense of guilt in children. These studies reported that a significant increase in the children’s distress was associated with a sympathetic reaction, which was reflected in a decrease in the temperature of the maxillary area and the nasal tip. A pleasant emotional state was analyzed by Nakanishi and Imai-Matsumura (2008), who observed 2- to 10-month-old infants when laughing. They reported a temperature drop in the nose area beginning at 4 months of age. As this drop was found by studies based on both stressful and pleasant situations, more research is needed to clarify the relationship between thermal and behavioral signals at an early age.

The present study was aimed at an investigation of thermal facial variations in 3- to 4-month-old infants involved in SFP. The main aim was to add to previous research by using a technique devised to maximize the preservation of the ecological context. According to thermal IR imaging literature that have indicated a decrease in facial skin temperature caused by stressful events, we expect to find such a decrease in the infants involved in SFP. If so, thermal data would confirm the higher activation of the sympathetic system compared to the parasympathetic during the still-face episode, as suggested by cardiac data.

## Materials and Methods

### Participants

Fifty consecutive mothers were contacted at the hospital of an urban area on the central-eastern coast of Italy within 2 days of their baby’s birth. All of them were requested to fill in a short questionnaire asking about demographic information and to participate in a study on infant development in the first 2 years of age. All mothers were given a detailed explanation about the study design and provided written informed consent according to the Declaration of Helsinki (World Medical Association Declaration of Helsinki, 1997). The protocol was approved by the local Ethics Committee of the University of Chieti-Pescara (Italy). Thirty-one mothers agreed to participate to the project. According to medical reports and maternal responses, no important differences were observed between the mothers who did and did not consent (with respect to race, nationality, language or clinical status). The mothers who did consent were contacted by telephone when their infant reached 3 months of age. On that occasion, they were invited to come to our University Baby Laboratory with their infant.

According to information provided by the mothers, all the 31 infants (16 males) were born full-term, none had medical complications at birth, or experienced hospitalisations or had been diagnosed with medical or psychological delays/disorders. They all belonged to bi-parental heterosexual Italian families, and their mothers were their primary caregivers. Seventeen out of the 31 dyads were excluded from the final sample for the following technical and procedural reasons: the cameras, either standard or thermal, stopped working, thus making the recording unavailable (*N* = 7); the mothers assumed facial expressions during the still-face episode, thus failing to meet the procedural requirements of the paradigm (*N* = 6); the infant turned his/her head to the side for a prolonged time, making it impossible to collect adequate frontal frames that were needed for detecting the facial temperature (*N* = 2); and experimental error (*N* = 2). Only two dyads were excluded, because the infants became too fussy at the very start of the first play episode to go on in the procedure. Therefore, the final sample included 12 infants (6 males) aged 3–4 months (age range: 2; 25–3; 21) and their mothers.

### Procedure

*Still-Face Paradigm.* The SFP (Tronick et al., 1978) was used to observe mother-infant interactions. This paradigm included three episodes: (1) the mother interacted with her infant in a playful manner, as she would usually do at home (*Pre Still-Face*; 3 min); (2) the mother became unresponsive, by assuming a neutral expression and maintaining a steady gaze at the infant, without touching or talking (*Still-Face*; 1 min); and (3) the mother reengaged face-to-face interaction with her infant (*Post Still-Face*; 3 min). As we used the SFP in a longitudinal project that was aimed at investigating the development of socio-cognitive understanding in the first 3 years of life, a fourth episode (*Objects*; 5 min) was introduced into the paradigm, during which the mothers played with their infant using a set of toys. The toys were provided by the experimenter and included a dog-shaped musical toy, a bell, a rattle, a soft bee-shaped toy, and a maraca. All but two of the mothers used the above toys over the entire episode. To specify the exceptions, the two mothers played with toys for approximately 2 min and 3 min, respectively, and interacted face-to-face during the remaining time. The experimenter provided a start signal for each episode. During the procedure, the infant was seated in an infant seat placed on a table facing his/her mother.

### Data Acquisition

The entire procedure was videotaped by a set of three cameras: one to record the infant, one the mother, and one both mother and infant. The films were subsequently mixed using the Avid Liquid 7 software to provide a three-split image. Subsequently, the films were processed in a video reading laboratory. Thermal IR imaging was performed using two digital thermal cameras (FLIR SC660, FlirSystems, Sweden), which were focused on the infant’s and the mother’s faces. The cameras were equipped with a Focal Plane Array of 640 × 480 detectors, 0.02-s time resolution, 0.03 *K* temperature sensitivity, and the capability of collecting the thermal radiation in the 7–14 μm band. The thermal camera response was blackbody-calibrated to null noise-effects related to the sensor drift/shift dynamics and optical artifacts. The sampling rate for thermal imaging was set at 10 frames/s. All of the observations were made in a climate-controlled room (room temperature: 23 ± 1 °C; relative humidity: 50–55%; no direct ventilation) located in a dedicated laboratory, and took place at approximately the same time of the day.

### Infant Interactive Behavior Coding

The infant’s behaviors were coded using the *Infant and Caregiver Engagement Phases* (ICEP; Tronick et al., 2005). The ICEP system includes a set of exhaustive and mutually exclusive infant and caregiver states, defined by configurations of facial expressions, direction of gaze, and vocalization. For the infant, the engagement codes are: *Protest*, *Withdrawn*, *Object/Environment Engagement*, *Social Monitor*, *Social Positive Engagement*, *Sleep*, and *Unscorable*. Additional codes aimed at capturing the infant’s regulatory behaviors are also included in the system, such as: *Oral Self-Comforting*, *Self-Clasp*, *Distancing*, and *Autonomic Stress Indicators* (for a description, see Table 1). To better analyze the infant’s attention to the environment, *Object/Environment Engagement* was distinguished into two separate codes: *Looking Around*, with the infant looking at the environment without focusing on any specific object; and *Object Engagement*, with the infant focusing on any object in the room for 1 s or more, both states were accompanied by non-negative expressions. During the first three episodes of the procedure, the objects included the infant’s hands, feet or clothing, and parts of the mother’s body (e.g., trunk, hands, jewelry), as well as the objects in the laboratory setting (cameras, one-way mirror, neon light, chair strap, side of the chair). In the final episode, the objects also included toys introduced by the mother.

**TABLE 1:** **Infant and caregiver engagement phases (ICEP; Tronick et al., 2005)**.

**Codes**	**Description**
*Protest*	The infant displays facial expressions of anger, grimaces, and/or is fussing, crying, arching her/his back, trying to get away, gesturing
*Withdrawn*	The infant is withdrawn and minimally engaged with the caregiver
*Object/Environment Engagement*	The infant looks at proximal or distal objects with an interested, neutral or positive facial expression, and may or may not vocalize
*Social monitor*	The infant looks at the caregiver’s face with a neutral or interested facial expression, and may vocalize in a neutral/positive manner
*Social positive engagement*	The infant looks toward the caregiver’s face with facial expressions of joy, including particularly smiles, and occasionally coos and play faces; the infant might vocalize in a positive manner, laughing, babbling, or squealing
*Sleep*	The infant is asleep
*Unscorable*	The infant’s face is obscured because of poor cameras angles or technical problems, or because the adult is blocking the camera focused on the baby
*Oral self-comforting*	The infant sucks on or brings to the mouth his/her thumb or wrist, something other than his/her body, and the mother’s hand or finger
*Self-clasp*	The infant’s two hands are touching
*Distancing*	The infant attempts to increase his/her physical distance from the caregiver without engaging an object
*Autonomic stress indicators*	The infant shows behaviors that might indicate stress or autonomic arousal, such as spitting up or hiccupping

Infant engagement phases and additional categories were coded every second from videotapes by a trained coder, using the Mangold Interact 8 software (version 8.1.3). Coding of an infant’s behavior was performed off-line using a frame-by-frame coding function (25 frames/s) with on/off activation of a keyboard key corresponding to the occurrence of each infant’s behavior code. An independently trained coder processed 25% of the sessions to compute inter-observer reliability. The resulting Kappa index for the engagement phases was 0.76. For the concurrent additional codes regarding the infant’s regulatory behaviors, the reliability index Interval by Interval, I × I (Birkimer and Brown, 1979), was 0.99 for Oral Self-Comforting and 0.98 for the Self-Clasp. The duration of each of the infant’s behavior codes was used for the analyses, and the proportion of time the infant was in each ICEP phase was computed by dividing the total time the code occurred in each episode by the total length of that episode.

As previously stated, mothers were provided with a signal for the start of each episode. However, it was possible that they needed a few seconds to respond. Therefore, during the coding phase, the start of the different episodes was determined according to a specific criterion, rather than on the experimenter’s signal: for the *still-face* episode, this corresponded to the first frame when the mother assumed a neutral and still expression; for the *post still-face* episode, to the first frame when the mother resumed face-to-face interaction; for the *objects* episode, to the first frame when the mother showed the toy to the infant. As *Withdrawn*, *Sleep*, *Distancing*, and *Autonomic Stress Indicators* were absent in our data, these codes were excluded from the analyses.

### Thermal Data Processing

The nose has been shown to be the most reliable region for the detection of psycho-physiological arousal (Ioannou et al., 2013). Therefore, in the present study, temperature observations were based on the nasal tip. Moreover, as the forehead turned out to be overall stress-insensitive in adults (Engert et al., 2014), that region has been considered as a potentially contrasting measure. The thermal videos were processed by a trained coder using IRI ImagePro software, a Matlab-based code for analysis of biomedical thermal imaging data, developed by the authors (Cardone and Merla) and validated in Manini et al. (2013). Specifically, to determine the infant’s facial thermal variations, the coder extracted one image/frame every 100 acquired frames, and detected the average temperature on two of the infant’s facial regions of interest, as the nasal tip and the forehead, selected according to previous studies with infant samples (Mizukami et al., 1987, 1990; Nakanishi and Imai-Matsumura, 2008; Ebisch et al., 2012; Ioannou et al., 2013; Manini et al., 2013). The coder made a circular marker on the regions of interest, and in each frame selected, an automatic tracking algorithm moved these markers to follow the same region across the images. To note, it was not always possible to respect the mentioned criterion for frame selection because during the procedure the infants were free to move and could turn their heads to the side, or their hands could hide their face, or the mother could block the camera that was focusing on the baby. In that case, the coder skipped to the next available frame with a reliable position of the infant. Finally, we obtained a pattern of the average temperatures of the nasal tip and the forehead across the episodes of the paradigm for each infant in the sample.

### Statistical Analysis

Statistical analyses were performed on both behavioral and thermal data using the SPSS software (IBM SPSS Statistics 19).

## Results

### Behavioral Data Analysis

Due to the skewed measures of infant behaviors, the data were analyzed using non-parametric tests. The Friedman overall test computed the differences in the duration of each code across the episodes of the procedure (pre still-face, still-face, post still-face, objects). Wilcoxon signed-rank tests were used to carry out pairwise comparisons (see Table 2). Results were significant for *Social Positive Engagement* [*χ*^2^ (3) = 16.22, *p* < 0.001], which consisted of gazing toward the mother accompanied by positive facial expression, and with possible vocalizations, which was higher in pre still-face than still-face, post still-face and the objects episodes (*Z* = 2.67, *p* < 0.01; *Z* = 2.07 and *Z* = 2.31, *p* < 0.05, respectively) and also higher in post still-face than still-face (*Z* = 2.37, *p* < 0.05). Significant results were also found for *Object Engagement* [*χ*^2^ (3) = 9.81, *p* < 0.05], which consisted of the gaze focused on any object in the room, which was higher in the final episode of the procedure when the mother introduced some toys into the interaction, compared to all three previous episodes (*Z* = 2.59, *Z* = 2.20, *Z* = 2.43, *p* < 0.05, respectively). *Looking Around*, although not significant at the Friedman test, it was significantly higher in still-face than in pre still-face episodes (*Z* = 2.40, *p* < 0.05). Also, although not significant at the overall test, *Self-Clasp* decreased significantly after still-face over the next two episodes (*Z* = 2.43, *Z* = 2.31, *p* < 0.05).

**TABLE 2:** **Means, standard deviations, and results of the Friedman tests and the Wilcoxon signed-rank tests that compared infants' behavioural responses across the episodes of the Still-Face Paradigm**.

**Behavior codes**	***M* (SD)**				**χ^2^**
	**Pre SF**	**SF**	**Post SF**	**Objects**
Looking around	0.07_*a*_ (0.09)	0.15_*b*_ (0.17)	0.07_*ab*_ (0.05)	0.06_*a*_ (0.06)	5.89
Object engagement	0.44_*a*_ (0.24)	0.38_*a*_ (0.32)	0.39_*a*_ (0.27)	0.72_*b*_ (0.17)	9.81*
Social monitor	0.39_*a*_ (0.23)	0.37_*ab*_ (0.40)	0.28_*ab*_ (0.23)	0.14_*b*_ (0.13)	3.50
Social positive engagement	0.07_*a*_ (0.07)	0.00_*b*_ (0.01)	0.04_*c*_ (0.06)	0.01_*bc*_ (0.03)	16.22***
Protest	0.01_*a*_ (0.02)	0.10_*a*_ (0.28)	0.14_*a*_ (0.31)	0.02_*a*_ (0.04)	3.96
Oral self-comforting	0.08_*a*_ (0.10)	0.12_*a*_ (0.18)	0.07_*a*_ (0.11)	0.05_*a*_ (0.06)	1.46
Self-clasp	0.22_*ab*_ (0.25)	0.34_*a*_ (0.31)	0.21_*b*_ (0.24)	0.18_*b*_ (0.22)	4.41

Mean values with differing subscripts within rows are significantly different at p < 0.05, except for the comparison between Pre still-face and still-face for Social Positive Engagement, which was significant at p < 0.01. SF, Still-Face. *p < 0.05, ***p < 0.001.

### Thermal Data Analysis

**FIGURE 1:**
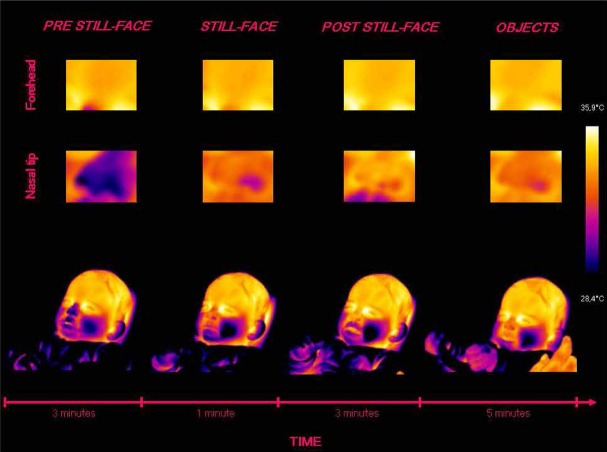
**Representative example of the variations in an infant’s facial temperature during the episodes of the Still-Face Paradigm**.

**FIGURE 2:**
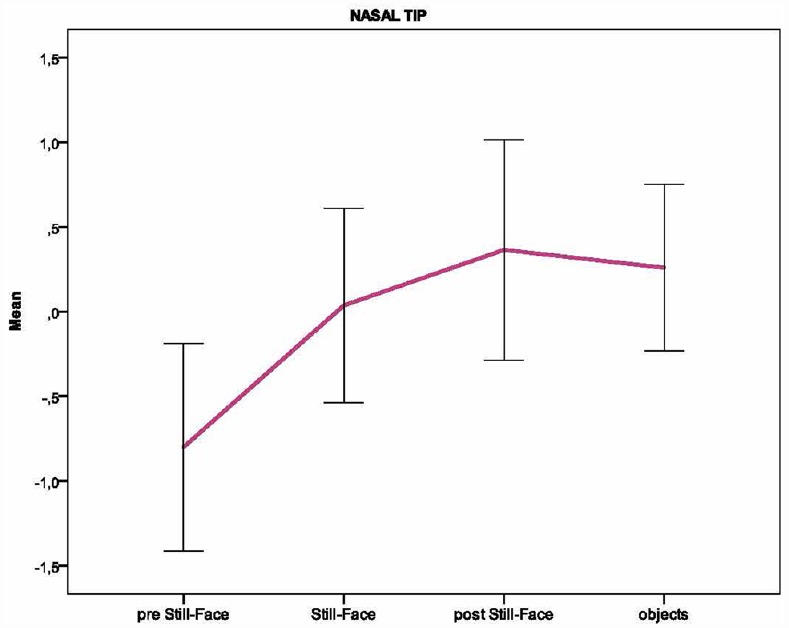
**Pattern of the temperature in the nasal tip across the episodes of the Still-Face Paradigm**.

**FIGURE 3:**
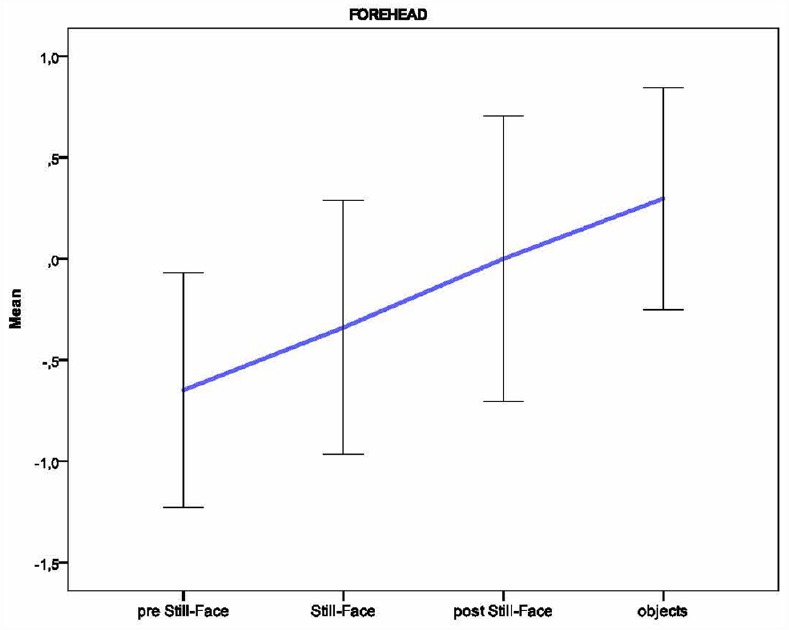
**Pattern of the temperature in the forehead across the episodes of the Still-Face Paradigm**.

Based on the individual average temperatures of the nasal tip and the forehead across the four episodes of the paradigm (for a representative example of the variations in an infant’s facial temperature, see Figure 1), a smoothing operation was performed that averaged five samples of temperature at a time, and the mean of the thermal signals which were previously transformed into z-scores, were calculated for each infant and episode. Friedman tests were performed separately on the mean nasal tip and the forehead temperature, with the episode (pre still-face, still-face, post still-face, objects) as a within-subjects factor. Pairwise comparisons between episodes were computed using the Wilcoxon signed-rank tests. Significant differences in nasal temperature emerged across the four episodes [*χ*^2^ (3) = 16.30, *p* < 0.001]. Specifically, the temperature increased sharply from the pre still-face to the still-face episode (*M_preSF_* = –0.80, SD_*preSF*_ = 0.61; *M_SF_* = 0.04, SD_*SF*_ = 0.57; *p* < 0.05) and remained significantly higher in the post still-face episode compared to the pre still-face (*M_preSF_* = –0.80, SD_*preSF*_ = 0.61; *M_postSF_* = 0.36, SD_*postSF*_ = 0.65; *p* < 0.05). Moreover, the nasal temperature increased from the still-face to the post still-face episode, although not significantly (*M_SF_* = 0.04, SD_*SF*_ = 0.57; *M_postSF_* = 0.36, SD_*postSF*_ = 0.65; *p* = ns; see Figure 2). The analyses of the forehead area showed variations in the temperature across the episodes [*χ*^2^ (3) = 14.50, *p* < 0.01]: this was significantly higher in the final than in the first episode (*M_preSF_* = –0.65, SD_*preSF*_ = 0.58; *M_obj_* = 0.30, SD_*obj*_ = 0.55; *p* < 0.05; see Figure 3).

There was little reason to affirm that each gradation of change in the skin temperature from play to still-face episode would be associated with a unique behavioral profile. However, a higher *versus* a lower thermal variation in the passage between the two episodes might be related to behavioral differences. Thus, to explore individual differences in behavioral responses to the still-face presentation based on thermal responses, we divided the infants into two groups (median split) based on the nasal temperature increase between play and still-face episodes. Specifically, all but one infant showed an increase in nasal temperature from the play to the still-face episode. Two results approached significance: infants who were above the median were higher in the composite category including *Looking Around* and *Object Engagement* compared to those who were below [*M* = 0.72 (0.32) vs 0.33 (0.43), respectively; *U* = 6, *p* = 0.065], whereas they were lower in *Protest* compared to the other group [*M* = 0.01(0.01) vs 0.20 (0.39), *U* = 7, *p* = 0.061].

## Discussion

The present study investigated the facial thermal responses in 3- to 4-month-old infants during the SFP, an experimental procedure designed to involve the infants in a perturbed face-to-face interaction. According to results reported in bio-behavioral research, which have shown that infant’s behavioral reactions to the still-face episode (i.e., positive behaviors decrease and negative ones increase) are accompanied by cardiac signals of distress (i.e., heart rate increases and vagal tone decreases), we expected to find stressful reactions at a thermal level; i.e., a decrease in facial skin temperature. This finding would confirm the prevailing activation of the sympathetic component of the autonomic system over the parasympathetic, which has been suggested to be the mechanism that mediates the infant’s autonomic reactions to the still-face presentation.

According to our behavioral data, *Social Positive Engagement* and *Looking Around* differed significantly in the still-face episode compared to the previous episode, with the former showing a decrease and the latter an increase. No significant differences were found between the two episodes for the remaining codes, such as *Protest*, *Object Engagement*, *Oral Self-Comforting*, and *Self-Clasp*, whereas *Withdrawn*, *Distancing and Autonomic Stress Indicators* were completely absent. Therefore, we confirmed the infants’ usual reactions to the still-face presentation with respect to social engagement (i.e., infants lowered their smiling, gazing, vocalizing to the mother while raising their attention to the environment), but failed to replicate normative data as to the aversive state (i.e., infants did not increase negative facial expressions or self-regulatory behaviors). Consistently, a carry-over of negative affect from the still-face episode was not found, as stressful behaviors were not more frequent in the reunion compared to the play episodes. Altogether, our data show that contrary to the report of the procedure as being distressing (Adamson and Frick, 2003), the infants we observed did not appear to be significantly disturbed by the maternal unresponsiveness.

The power of SFP to upset infants has been considered in the literature (Adamson and Frick, 2003; Mesman et al., 2009). Based on the relative lack of crying in the still-face episode (Adamson and Frick, 2003), the short-term maternal unresponsiveness caused by the paradigm was thought of as presenting the infants with a sobering or mild social stressor, which had the potential to amplify their tendency to become distressed (Tronick et al., 1978; Toda and Fogel, 1993; Moore and Calkins, 2004). Therefore, negative responses are expected to vary, based on the impact that an event has on the infant’s affective state. Indeed, different reactions did result from descriptions of the still-face effect, as related to individual dispositions (Stoller and Field, 1982; Gianino and Tronick, 1988; Ham and Tronick, 2006; Ekas et al., 2013), developmental processes (Toda and Fogel, 1993), or group characteristics (Tronick, 2003). As variations in the paradigm (e.g., altering the order of the conditions, substituting the mother with a stranger, or touching the infants by a still-face adult) also proved to affect the extent to which the still-face episode was stressful (for a review, see Mesman et al., 2009), a procedural detail such as the duration of that episode would be a possible influencing factor. As documented by the meta-analysis reported by Mesman et al. (2009), still-face duration ranges from 30 s to 180 s across studies, and although its influence on the degree of still-face effect has not been explicitly tested, longer durations are expected to cause longer perturbations, and thereby to have larger negative effects. It might be that the perturbation time we adopted in the current study (i.e., 1 min) was long enough to make the infants aware of the removal of the maternal attention, but too short to stress them to any relevant level. That is, it caused the infants to withdraw from interacting and to shift their attention toward the environment, without significantly modifying their affective state with respect to the previous non-perturbed period. If so, infants involved in our experimental procedure would be likely to find the still-face episode a novel rather than a distressing event.

This result indicates the infant’s recognizing the interruption of the interactional reciprocity as a relevant factor for the still-face effect. The infants changed their behavior in accordance with the change in the maternal interactive style, lowering their communicative bids, and turning their attention to the outside environment, while remaining calm throughout the procedure (for a similar result, see the “Stably Low” subgroup of infants in Ham and Tronick, 2006). Even if the still-face effect “does not admit to a simple explanation” (Adamson and Frick, 2003, p. 464) and indeed, according to Tronick’s reconceptualization (Tronick, 2003) of the nature of that effect, any attempt to explain this effect must go beyond the infant’s cognitive reactions to the perturbed situation, our results support the recent recommendation of Mesman et al. (2009, p. 156) that the role of an infant’s expectations about the nature of social interactions should not be ruled out entirely from an explanation of the still-face effect. Indeed, the infant’s sensitivity to changes in social contingency during early face-to-face interactions has largely been proven (e.g., Bigelow and DeCoste, 2003). Consistent with this, other literature has clarified their ability to capture regularities in the environment based on spatio-temporal rules: they are able to extract information from the statistical properties of auditory events, such as the sound sequence in familiar words (Saffran et al., 1996); to detect the sequential order in a series of visual events, to develop expectations for these events, and to act on the basis of these expectations (Haith et al., 1988); and to react to the newly formed expectations being violated (e.g., Shapiro et al., 1998). Therefore, as in the Frick and Adamson (2003) conclusion, it is plausible that infants refrain from their communicative attitude when the usual interactions are perturbed. Whether they also become distressed by that perturbation would depend on the impact that this event has on the infant psycho-physiological functioning.

Thermal data were in line with the above behavioral pattern, as facial skin temperature did not decrease in the still-face episode compared to the pre still-face. Under the assumption of SFP as a stressful event, this result was not expected, as a drop in facial skin temperature usually accompanies stressful situations as an effect of the sympathetic activation (Mizukami et al., 1987, 1990; Levine et al., 2001; Nakayama et al., 2005; Merla and Romani, 2007; Shastri et al., 2009; Kuraoka and Nakamura, 2011; Ebisch et al., 2012; Ioannou et al., 2013; Manini et al., 2013; Di Giacinto et al., 2014; Engert et al., 2014; Merla, 2014). However, the negativity did not increase during the still-face episode, and thus the sympathetic component was not active, and consequently, there was no decrease in facial skin temperature. The absence of sympathetic activation signaled by thermal data appears to be at odds with the cardiac evidence provided by previous studies; e.g., an increase in heart rate and a decrease in RSA, as according to the Polyvagal Theory (Porges, 2007), both outputs would signal the lowering of the parasympathetic component to potentiate the sympathetic. However, if we consider the low impact that the still-face presentation had on the infant’s affective functioning in our sample, all of the measures were consistent in revealing the nature of infant’s autonomic regulatory system as related to an appropriate reactivity. Altogether, the cardiac and thermal results show that when the SFP causes the infants to be distressed, as in previous studies, the sympathetic component of that system is active. When it causes a lower stressful effect, as in the present study, activation of that component is lacking.

Unlike the sympathetic component, the parasympathetic was active. As we found, the facial skin temperature rose on the nasal tip from the pre still-face to the still-face episode. Indeed, it was also higher in the forehead area during the objects episode than in the pre still-face. Therefore, a thermal variation was detected during the procedure, which suggests that a change occurred in the infant’s autonomic system. As that variation consisted of a thermal rise, it would signal an activation of the parasympathetic component. According to the Polyvagal Theory (Porges, 2007), this component mediates the infant’s positive engagement with persons and objects. A number of results support that claim. Huffman et al. (1998) provided indirect evidence of the association between vagal activity and the state of engagement when they showed that regulation of the vagal brake during a challenging task is related to the temperament dimension of orienting, as measured by the mothers filling in of the Infant Behavior Questionnaire (Rothbart, 1981). More directed evidence was found in other studies. Bazhenova et al. (2001) compared the infant’s vagal regulation during the still-face procedure and the object activity, and showed a decrease in RSA during the former and an increase during the latter, which varied in parallel with the infant’s state of attention. A similar result was obtained in a subsequent study (Bazhenova et al., 2007) that compared the RSA amplitude in two situations, when the researcher was looking at the infant while also smiling, and when the researcher was only looking: increases in RSA were observed under the latter condition relative to the former condition. Altogether, these results suggest that when the infants are faced with an object or a non-responsive adult in a context that lacks negativity, vagal activity allows them to maintain an active, however, calm, state, in order to search for relevant cues for engaging with objects and people. Consistent results were also reported in a previous study (DiPietro et al., 1992), where the infant’s autonomic reactions to a surprising event were measured (i.e., Jack-in-the-Box presentation). This study showed that infants who increased in RSA during the stimulus presentation responded with longer bouts of engagement with objects, compared to infants who showed a decrease in RSA. Again, the infant’s interest in object exploration and the parasympathetic activation were closely related. This link has been confirmed by a stream of studies on early attention development (see Richards, 2001; Courage et al., 2006; Richards et al., 2010). In particular, Richards and Casey (1991) showed that heart rate changes during stimulus presentation paralleled the depth of the attentive arousal: at the onset of the presentation, the heart rate showed a large, rapid deceleration from the prestimulus level, which was maintained subsequently, when the infants were visually processing the stimulus. Heart rate returned to the prestimulus level in the phase of attention termination. Therefore, conditions of attentive arousal, which correspond to the first two orienting and sustained attention phases, were mediated by the parasympathetic nervous system, which acted to slow down the heart rate (Richards and Casey, 1991).

In the light of previous studies, the parasympathetic activation we found during the SFP might be related to the infant’s attention to the environment. Indeed, the thermal rise in nasal tip during the still-face compared to the pre-still face episode was accompanied by a significant increase in the infant’s looking around, which in still-face episode was twice that in the previous episode [*M*(SD)_*preSF*_ = 0.07(0.09), *M*(SD)_*SF*_ = 0.15(0.17); *p* < 0.05]. Also, the thermal rise for the forehead during the last episode, when an object was made available for exploration, compared to the first episode, went along with a high level of engagement with that object. Results at the individual level also showed a relationship between thermal and attention measures. They only approached significance in this case, therefore signaling a tendency rather than any clear effect. However, they went in the same direction as the significant results just discussed, and a larger sample would probably provide a more robust result. According to our data, infants who were above the median value of thermal increase between the play and still-face episodes were higher in the engagement with the environment (a composite category including *Looking Around* and *Object Engagement*) during the still-face episode, compared to the infants who were below. Moreover, the “above” infants were lower in *Protest* during the same episode than the “below” infants (for a change in protest-heart rate measures also based on group membership classification, see Ham and Tronick, 2006). Interestingly, no relevant differences emerged between the two groups with respect to any of the other variables. Therefore, parasympathetic activation, as signaled by the facial temperature increase in the still-face episode compared to the pre still-face, was shown to be related to the infant’s involvement with the physical environment. Moreover, infants who were higher in parasympathetic activation were more involved, compared to the infants who were lower, and at the same time they experienced less negativity.

## Conclusion

To our knowledge, this is the first study that has examined thermal correlates of the still-face effect. We have shown that the facial skin temperature rose significantly from the pre still-face to the still-face episode, thus signaling a lack of sympathetic activation and an increase in the parasympathetic activation. We also showed a decrease in the communicative signals and an increase in the attention to the environment, with no signals of distress. Therefore, with reference to the debate about the nature of the still-face effect, behavioral and thermal data are consistent in supporting an interpretation of the infant’s adjustment to the maternal unresponsiveness as closely related to the infant’s recognizing that the usual way of interacting has been disrupted. The present study is also the first that reveals an association between attention and facial skin temperature. We showed an activation of the parasympathetic component of the autonomic system associated with an increase in the infant engagement with the environment. As this result is consistent with research using cardiac measures, the thermal IR imaging was reliable for detecting the infants’ autonomic variations not only in the emotional system, as in other studies conducted to date, but also in the attention system.

The limitations of the present study relate primarily to the small sample size, which was reduced from the initial sample due to technical and procedural reasons. This prevented us from reaching more robust conclusions about the relationships between the infants’ thermal reactions and their behavioral responses during the still-face presentation. Although small, our sample has provided data that are very consistent between these two levels of observation. They show a bio-behavioral pattern that is revealing in terms of the possible factors that underlie the still-face effect, thus contributing to elaboration of that effect at a theoretical level.

Another limitation is more general and relates to the difficulties in the interpretation of physiological data. Having concurrent and more canonical measures of the autonomic system variations to the still-face procedure would have allowed this difficulty to have been partly managed. However, we chose to record autonomic reactions in an ecological context, thus avoiding physiological procedures, which, as for those available to date, require manipulation of the infant’s body in a more or less invasive manner. We hope that devices for recording autonomic signals improve their feasibility, thus becoming more friendly and easier to use in developmental research.

## Author Contributions

TA, AG are responsible for the conception of the study, the acquisition, analysis and interpretation of the behavioral data, and the drafting of the manuscript; DC, AM are responsible for the acquisition, analysis and interpretation of the thermal data, and the drafting of the manuscript.

### Conflict of Interest Statement

The authors declare that the research was conducted in the absence of any commercial or financial relationships that could be construed as a potential conflict of interest.
